# Experimental Evaluation of Tensile Behavior and Hygrothermal Degradation of Glass Fiber Composites

**DOI:** 10.3390/polym18020277

**Published:** 2026-01-20

**Authors:** Ciprian Ionuț Morăraș, Viorel Goanță, Lucia Raluca Maier, Teodor Adrian Badea, Paul Doru Bârsănescu

**Affiliations:** 1 Mechanical Engineering, Mechatronics and Robotics Department, Mechanical Engineering Faculty, “Gheorghe Asachi” Technical University of Iasi, 700050 Iasi, Romania; viorel.goanta@academic.tuiasi.ro (V.G.); paul-doru.barsanescu@academic.tuiasi.ro (P.D.B.); 2 Composite Materials Laboratory for Aeronautical Field, Romanian Research & Development Institute for Gas Turbines—COMOTI, 220D Iuliu Maniu Av., 061126 Bucharest, Romania; teodor.badea@comoti.ro

**Keywords:** glass fiber-reinforced polymer, tensile test, impact test, immersion, mechanical properties, DMA, TMA

## Abstract

Glass fiber-reinforced polymer (GFRP) composites are widely used in structural applications due to their high specific strength and durability; however, their mechanical performance strongly depends on fiber architecture and environmental exposure. This study evaluates the mechanical behavior and moisture-induced degradation of GFRP laminates through tensile tests, impact tests, dynamic mechanical analysis (DMA), and thermomechanical analysis (TMA) performed on a bi-directional glass–epoxy GFRP laminate ([0°/90°]). Tensile tests revealed a maximum longitudinal strength of 369 MPa in dry specimens, while water immersion for up to 21 days led to a significant reduction in tensile strength, from 207 MPa to 63 MPa, in diagonally cut specimens. Impact tests conducted at 12 J showed larger displacements in specimens cut along directions not aligned with the fibers, indicating matrix-dominated behavior. Dynamic mechanical analysis demonstrated strong dependence of stiffness on fiber orientation, with storage modulus values decreasing by approximately 45% in 45° specimens compared with the principal directions, while the glass transition temperature remained within 59–62 °C. Thermomechanical analysis confirmed an increase in the coefficient of thermal expansion after aging, from 205.6 to 291.65 µm/(m·°C) below Tg. These results provide insights into the structure–property–environment relationships governing the durability of GFRP composites and support the optimization of their design for long-term polymer-based applications.

## 1. Introduction

Glass fiber-reinforced polymer (GFRP) composites are increasingly used in engineering applications due to their high strength-to-weight ratio, corrosion resistance, durability, impact resistance, and relatively low cost [1]. Their mechanical performance is strongly influenced by fiber type, architecture, orientation, volume fraction, and manufacturing techniques, making the selection of optimal material configurations essential to specific industrial applications [2,3,4,5,6]. Owing to the structural complexity of composite materials, numerous studies have reported a strong correlation between fiber morphology and distribution, fiber–matrix interaction, and the viscoelastic behavior of the polymer matrix. A plain-textile-reinforced GFRP composite (RT500) exhibited a longitudinal Young’s modulus of 21,337 MPa, approximately double that of the MAT450 composite (10,238 MPa), as well as showing approximately 52% higher tensile strength and maintaining superior performance under compression [7,8,9]. The mechanical and elastic properties of GFRP composites are largely determined by the modulus of the fibers, their strength, and the effectiveness of the interaction at the matrix–fiber interface [10,11,12]. To validate the capability of these materials to withstand complex loads in engineering applications, they must be subjected to an extensive range of mechanical tests, both static (tension, compression, shear, and bending) and dynamic (fatigue), in accordance with the mechanical stresses occurring during service. Studies have shown that mechanical properties—such as tensile strength, flexural strength, and impact strength—can vary significantly depending on the type of polymer matrix used in GFRP composites [13]. Another study [14] shows that epoxy-based resin matrices are valued for their superior strength and stiffness, which makes them suitable for applications requiring high mechanical performance. In addition to mechanical properties, the choice of polymer matrix can also influence other factors, such as thermal stability, chemical resistance, and moisture absorption in GFRP composites [15].

In the study in [16], the effect of fiber loading and fiber orientation on tensile behavior, and the impact strength of polymer composite materials was investigated. The results show that increasing fiber loading enhances the strength of the polymer composite and also affects the mechanical behavior and corrosion resistance of reinforced composites. Additionally, the strength and stiffness properties increase proportionally with the fiber volume fraction in composites. Beyond mechanical properties, the thermal conductivity of the composite material also improves by approximately 10% as the fiber volume fraction increases.

Environmental effects such as temperature variations, moisture exposure, and aging significantly accelerate the degradation of GFRP composites by weakening the fiber–matrix interface and altering the viscoelastic response of the matrix [17,18,19,20,21,22]. Three types of fiber-reinforced polymer composites were analyzed by Selzer and Friedrich [23] to assess the impact of moisture on their mechanical properties and fracture behavior. The study used two thermoset matrices (unmodified epoxy and toughened epoxy) and one thermoplastic matrix (polyetheretherketone). The authors observed a reduction in these properties as a result of moisture absorption, which they attributed to the weakening of the fiber–matrix interface and the softening of the matrix materials.

Gu Huang et al. [24] observed that immersing glass fiber composites in water for periods of 7, 14, and 21 days leads to water absorption due to the capillarity of the material and the hydrophilic groups present in both the fibers and the matrix. This causes degradation at the fiber–matrix interface and significantly reduces mechanical strength. In the case of the dry sample, the tensile strength was 192 MPa, and for the sample immersed for 21 days, it was 162 MPa. Other studies, such as [25,26], have observed that reducing the glass transition temperature, Tg, below water absorption leads to plasticization, swelling, matrix hydrolysis, mass loss, and delamination of the fiber–matrix interface of the composite. It is known from the literature that the mechanical properties of GFRP degrade at high temperatures, especially near the glass transition temperature (Tg) of the polymeric matrix (approximately 65–150 °C). Thus, the tensile strength of GFRP decreases by about 40% in this temperature range compared with the tensile strength at 20 °C [27].

Some research papers, such as [28,29], analyzed the impact behavior of two types of GFRP composites, C-600 and E-800, made from C-glass–epoxy (600 g/m^2^) and E-glass–epoxy (800 g/m^2^). The tests were carried out using eight impact energy levels ranging from 6 J to 48 J. The experimental results showed that for both GFRP materials, increasing the impact energy produced a clear and direct correlation with the intensification of damage and the variation in impact parameters (increased displacement, changes in maximum force, and absorbed energy). The differences between the materials—particularly the thickness determined by the number of layers and their intrinsic mechanical properties—had a significant influence on the impact response. Due to its higher specific mass and increased rigidity, GFRP E-800 demonstrated superior resistance compared with GFRP C-600, maintaining a higher capacity to withstand loads and limit damage at the same energy levels.

This study provides an evaluation of a GFRP laminate commonly used in wind turbine blades, making contributions to the understanding of moisture-induced degradation and the mechanical behavior of GFRP composites. Tensile tests allowed for a more accurate assessment of orthotropy and its evolution. Water immersion for 7, 14, and 21 days, combined with impact testing and DMA/TMA analyses performed on both dry and aged specimens, enables a deeper understanding of how moisture influences stiffness, viscoelastic properties, and thermomechanical stability. Overall, this study provides an application-oriented experimental contribution, supporting improved durability assessment and structural reliability of GFRP components used in wind energy systems.

## 2. Materials and Methods

### 2.1. Materials

The glass–epoxy composite laminate was manufactured by vacuum-assisted RT lay-up from 10 plies of bi-directional plain weave-woven glass (areal weight of 290 g/m^2^) impregnated with a mixture of IN2 Epoxy Infusion Resin based on bisphenol-A-(epichlorhydrin) reaction product and an “slow” amine hardener in a 1:2 proportion. The laminate was 4 mm thick, and the fiber orientation was [0°/90°]. The characteristics of the resin are presented in Table 1 [30]. The fiber volume of the studied composite was 42%.

**Figure 1:**
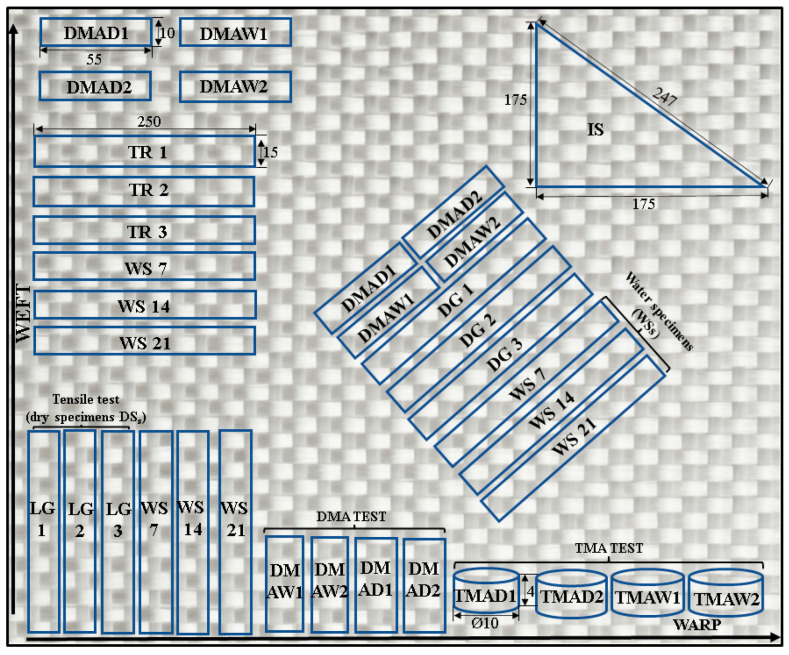
Cutting directions for tensile test, DMA, TMA and impact test.

Figure 1 shows the set of specimens and the cutting direction for each test. In the case of the tensile test, specimens were cut in 3 directions: three specimens in the longitudinal direction 0° (denoted LG1-LG3), three in the transverse direction 90° (denoted TR1-TR3) and three in the diagonal direction 45° (denoted DG1-DG3). In the case of the aged specimens, three specimens were tested in tension in the 3 cutting directions (denoted WS7, WS14, and WS21). For the DMA tests, two specimens were tested in the 3 cutting directions (aged and unaged, denoted DMAW1-2 and DMAD 1-2). For the TMA test only in the longitudinal direction, two specimens were tested (aged and unaged, denoted TMAW 1-2 and TMAD 1-2). The triangular specimen was used for the impact test in the three directions.

### 2.2. Experimental Setup

#### 2.2.1. Tensile Tests

**Figure 2:**
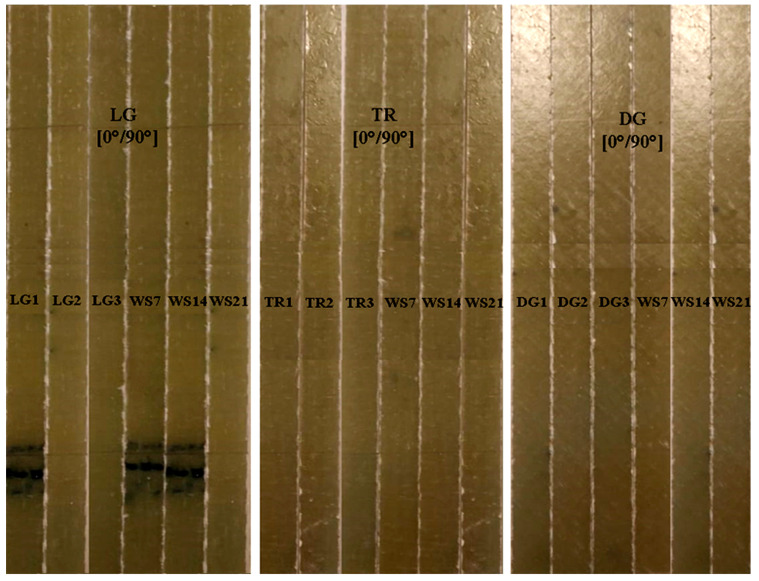
Set of specimens used for the tensile and aging conditioning tests, according to ASTM D3039 [31].

Tensile test specimens were prepared according to the ASTM D3039 standard in order to determine the material characteristics [31]. In this study, to evaluate the mechanical behavior of the GFRP composites, the tests were performed using a WDW50 universal mechanical testing machine manufactured by Jinan Hensgrand Instrument Co., Ltd. (Jinan, China). The equipment has a maximum load of 50 kN and a testing area of 600 × 600 mm^2^, with a measurement range between 0.4% and 100% of the full scale (FS). The machine is operated through an automated closed-loop AD800 measurement and control system. The dedicated software tool (WinWdw Electronic Universal Testing Machine Measure and Control System, version 2.15.P.E) was used for data acquisition and control. The specimens were loaded at a constant speed of 0.5 mm/min until breaking. Figure 2 shows the set of specimens used for the tensile test in the three cutting directions (TR, LG, and DG).

Water absorption tests were conducted in accordance with ASTM D5229 [32]. Prior to immersion, the specimens were weighed using a Partner analytical microbalance with a resolution of 1 mg to determine the initial mass *m*_0_. The samples were then fully immersed in distilled water at ambient temperature for 7, 14, and 21 days (WS7, WS14, and WS21) for all three cutting directions (LG, TR, and DG). After each conditioning period, the specimens were removed, gently surface-dried using absorbent paper, and immediately weighed to determine the conditioned mass m_t_. The water absorption content was calculated as the percentage mass gain relative to the initial mass, using Equation (1):

(1) $$M(\%)\;=\;\frac{m_{t}\;-\;m_{0}}{m_{0}}\times 100$$

Based on the results obtained after the failure of the specimens, the stress–strain curves were generated. Considering the relatively high variability of the experimental data, a statistical analysis was also performed in order to establish the required characteristic values.

#### 2.2.2. Three-Directional Impact Test on a GFRP Sample Using a Drop Weight Machine

Wind turbine blades are constantly exposed to environmental conditions: humidity variations, temperature variations, snow, and impact from wind-borne particles (sand, pebbles, and hail). It is known from the literature that most wind turbine blades are made of GFRP, a material that absorbs water, which leads to a decrease in the mechanical and elastic characteristics of the material. This paper studies the influence of humidity and impact on GFRP materials. The ASTM D7136 standard [33] specifies impact on the center of a composite plate, measuring 150 mm × 100 mm, embedded on the contour. In the case of wind turbine blades, hail or wind-borne pebbles can cause damage by impacting the leading and trailing edges of the blades. The ASTM D7136 standard [33] does not reflect the effect of these impacts, the influence of the test piece edges, and the reinforcement directions. This was studied in the paper, proposing a triangular impact test piece instead of a rectangular one as in ASTM D7136.

**Figure 3:**
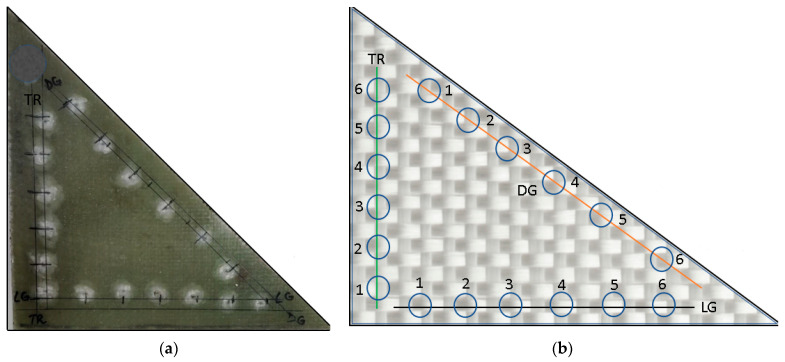
Sample used and impressions resulting from indentation: (**a**) Impacted sample. (**b**) Arrangement of indentations.

Impact tests were performed on a single triangular GFRP specimen (the specimen was cut from the plate presented in Section 2.1), as shown in Figure 3a. The tested specimen was not subjected to any additional immersion or aging processes.

In the case of a drop test, the instantaneous contact force is given by the relationship

(2) $$F\left(\delta \right)=k\left(\delta \right)\cdot \delta$$

where

- F is the impact force;
- Δ is the displacement/indentation;
- $k\left(\delta \right)=\frac{dF}{d\delta \;}$ is the contact stiffness (which is nonlinear for GFRP).

When a mass *m* falls from a height h, upon the release of all energy to the sample when the maximum displacement δ_max_ is recorded, we will have

(3) $${\;\delta }_{max}=\sqrt{\frac{2mgh}{k}}$$

where

(4) $$\;F_{max}=k\cdot \delta_{max}$$

After loading, the sample will undergo a total displacement composed of an elastic displacement δ_e_ (with return) and a residual displacement δ_p_:

(5) $$\delta_{max}=\delta_{e}+\delta_{p}$$

The elastic recovery ratio is defined as

(6) $$R_{e}=\frac{\delta_{e}}{\delta_{max}}$$

For GFRP, when R_e_ is high, we have a pronounced elastic response, and when R_e_ is low, the sample suffers large internal damage.

Testing was carried out at low impact velocities and with a relatively small drop mass (8.2 kg), using a hemispherical indenter with a diameter of 10 mm. A total of 18 impacts were performed, six on each side of the triangular specimen used, as shown in Figure 3b. The directions in which the indentations were made were designated as longitudinal (LG), transversal (TR), and diagonal (DG), with respect to the reinforcement direction presented in earlier chapters. All impacts were conducted with the same energy (the same mass dropped from the same height), namely, 12 J, with the only difference between the tests being the change in the impact location.

**Figure 4:**
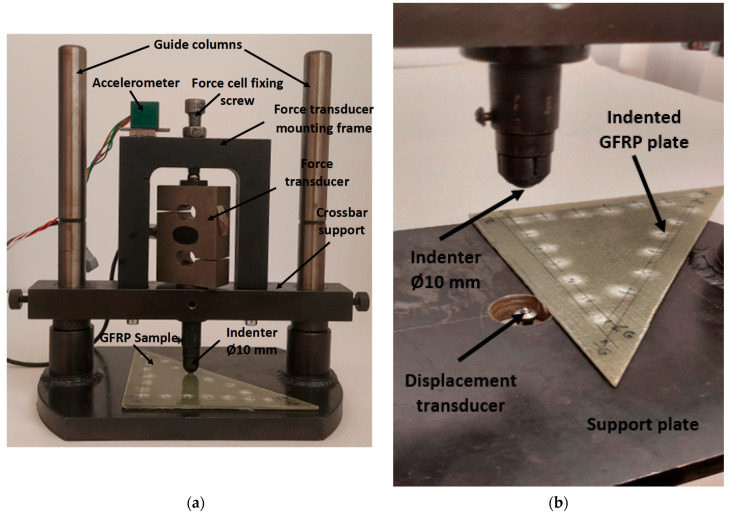
Experimental assembly used for drop test: (**a**) Equipment components. (**b**) Location of the indenter and displacement sensor.

As can be seen in Figure 4a, three sensors were mounted on the test stand and are described below. The force sensor can be used up to 20 kN with a transformation coefficient of 2 mV/V at maximum force. The displacement sensor, shown in Figure 4b, can be used up to 5 mm with a transformation coefficient of 3.536 mV/V up to maximum displacement. The power supply of these sensors was under an electrical voltage of 4 V. The accelerometer was of the ADXL377 type (Analog Devices, Wilmington, MA, USA), with an acceleration range of ±200 g and a sensitivity of 6.5 mV/g at 3 V. The signals from the force and displacement sensors, as well as the acceleration signal in the vertical direction (Z), were acquired with an NI 6009 acquisition board (National Instruments product, Austin, TX, USA). Considering the shock loading to which the GFRP composite plate was subjected, the acquisition rate was set to 1000 Hz for all three signals.

#### 2.2.3. Dynamic Mechanical Analyzer (DMA)

Dynamic mechanical analysis (DMA) was conducted on a TA Instruments DMA 850 analyzer (New Castle, DE, USA) and working in three-point bending mode with a 1 Hz load frequency of oscillation and ±1 modulus precision according to the ASTM D7028-7:2015 standard [34]. The test was run at a 5 °C/min rate from room temperature to 70 °C. DMA tests are individual measurements with an isothermal stability of ±0.1 °C above 50 °C. The tan δ resolution and tan δ sensitivity of TA DMA 850 are 0.00001 and 0.0001, respectively. The shape of the specimens was rectangular, with dimensions of 55 mm, 10 mm, and 4 mm. A set of 2 samples was utilized for each test (dry and aged). The samples were cut in 3 different directions, LG, TR, and DG, from the epoxy–glass fiber-reinforced laminate manufactured as described above, in Section 2.1.

Dynamic mechanical analysis (DMA), by depicting the storage modulus, loss modulus, and loss factor (damping) as a function of temperatures, characterizes the temperature dependency of intrinsic mechanical properties for viscoelastic materials.

#### 2.2.4. Thermomechanical Analysis (TMA)

The coefficient of linear thermal expansion was measured using the TMA 450 (New Castle, DE, USA) thermomechanical analyzer by TA Instruments. The tests were carried out in the temperature range from room temperature up to 70 °C. The heating rate of the samples was 5 °C/min. The samples were tested in the warp direction. The shape of the specimens was circular, with a diameter of 10 mm, according to ASTM E831-24 [35]. A set of 2 samples was utilized for each test (dry and aged). The samples were cut in the LG direction, as described above, in Section 2.1.

TMA analysis measured dimensional changes with temperature to determine the coefficient of linear thermal expansion (CLTE) and the glass transition temperature (Tg). Based on the following equation, Equation (7), the coefficient of thermal expansion was calculated:

(7) $$\;\;\;\;\;\;\propto \;=\;\frac{x-x_{0}}{x_{o}∆T}=\frac{∆x}{x_{0}∆T}$$

where *x*—sample length after temperature change; $x_{0}$—initial length; α—coefficient of thermal expansion; and ΔT—temperature increase.

## 3. Results

### 3.1. Mechanical Properties of GFRP

**Figure 5:**
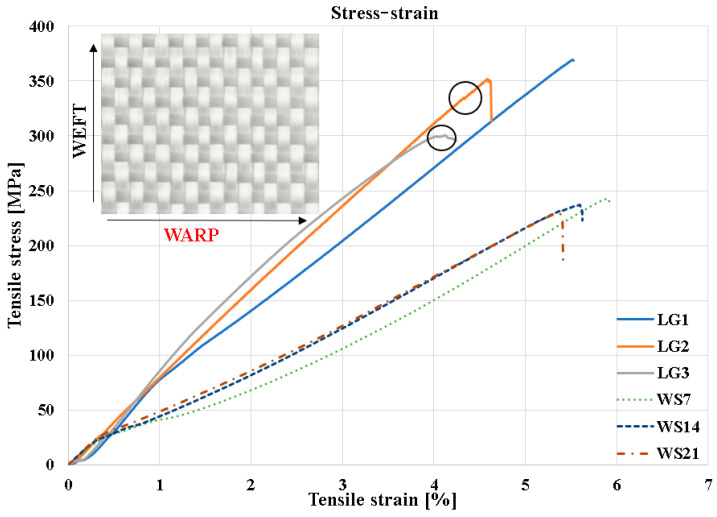
Stress–strain diagrams for specimens with fiber orientation of [0°/90°]. The circles in the figure represent the moment when the initiation of the interlaminar microcrack begins.

Figure 5 represents the superimposed stress–strain curves of the samples cut in the longitudinal direction (LG1–LG3), as well as for those immersed for 7, 14, and 21 days. By comparing the three curves, it can be observed that the highest failure stress occurs in specimen LG1, with a maximum value of σ_r_ = 369 MPa. By analyzing the stress–strain characteristic curves, it can be observed that between the elastic limit and the final failure zone there is an initial segment that reflects the interlaminar behavior and the response of the outer lamina during loading (most of the tensile load is carried by the fibers oriented in the direction of the applied force). The onset of failure is marked by the appearance of interlaminar microcracks in the material, which produce abrupt changes in the characteristic curve, clearly visible in specimens LG2 and LG3. Once the interlaminar microcracks are initiated, the tensile stress is redistributed to the remaining structure of the composite. This phenomenon leads to the rapid propagation of damage and ultimately to the fracture of the composite. In specimen 2, the first microcrack initiated at a stress of approximately σ_r_ = 340 MPa, while in specimen 3, it initiated at around σ_r_ = 300 MPa. A similar behavior for plain weave reinforced composites has been reported in [8,36,37,38]. It was also found that the slopes of the three stress–strain curves indicate low energy storage capacity during deformation, a characteristic feature of materials exhibiting viscoelastic behavior.

The mechanical behavior of the specimens immersed in water for 7, 14, and 21 days (WS7, WS14, and WS21) shows progressive degradation of the mechanical properties compared with the dry specimens (LG1–LG3). The stress–strain curves of the conditioned samples exhibit a lower initial slope, indicating a reduction in stiffness and a decrease in Young’s modulus due to moisture absorption within the polymer matrix and at the fiber–matrix interface. Moreover, the maximum tensile strength decreases significantly with the increase in immersion time, confirming the impairment of the composite’s ability to transfer load to the fibers. Unlike the dry specimens, which display noticeable jumps in the characteristic curves caused by the initiation of interlaminar microcracks, the immersed samples show a more uniform deformation response, typical of a material with a softened matrix and a weakened interface. Therefore, prolonged exposure to a humid environment leads to clear degradation of the mechanical behavior.

**Figure 6:**
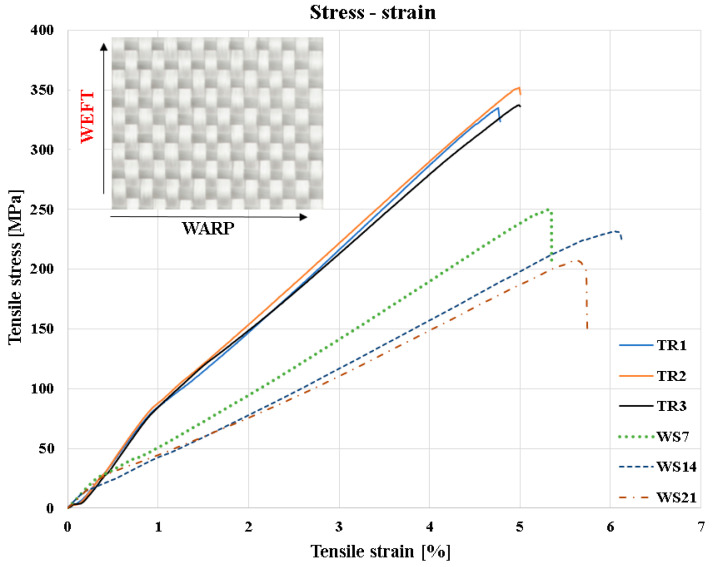
Stress–strain diagrams for specimens with fiber orientation of specimens TR [0°/90°].

In Figure 6, the superimposed stress–strain curves are presented. The transverse specimens (TR1–TR3), oriented in the weft direction, present lower strength values and a smaller initial slope, indicating a reduced contribution of the fibers to load taking and a more pronounced influence of the polymer matrix on the deformation behavior. The mechanical behavior of the aged specimens in this direction shows progressive degradation of mechanical performance, evidenced by a pronounced reduction in stiffness and in the maximum tensile strength. The gradual decrease in the slopes of the curves confirms the softening of the polymer matrix and the weakening of the fiber–matrix interface, effects that become more pronounced with the increase in immersion time.

**Figure 7:**
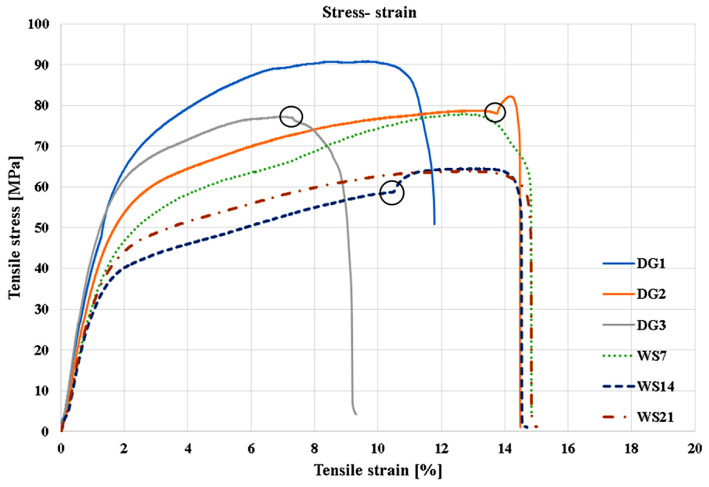
Stress–strain diagrams for specimens with fiber orientation of specimens DG [0°/90°].

The overlapping stress–strain curves in Figure 7 show significant differences in tensile strength behavior for the three specimens cut at 45° with respect to the plate with fiber orientation of [0°/90°], as well as for the aged specimens. In the case of the specimens cut at 45° (DG1–DG3), the loading is no longer applied along the principal fiber directions; the mechanical response is, therefore, governed primarily by the matrix and by in-plane shear mechanisms within the laminate. This leads to a pronounced deviation from linearity in the stress–strain curves and to significantly lower tensile strength values compared with the longitudinal and transverse specimens.

The specific elastic energy stored in the unit volume of the dry and wet specimens was calculated. It was found that wet specimens stored a specific elastic energy of about 27.4% of the specific energy stored by the dry specimens.

The stress–strain curves of the DG specimens exhibit very large strains at failure and an extended elasto-plastic region, indicating an elasto-plastic behavior characteristic of materials in which the matrix carries the majority of the applied load. This mechanical response is a direct consequence of the 45° orientation, a direction in which the fibers do not significantly contribute to load bearing and where the deformation is dominated by the properties of the polymer matrix and by the strength of the fiber–matrix interface. The stress–strain curves for the aged samples (WS7, WS14, and WS21) show progressive deterioration of mechanical properties with the increase in immersion time. The initial stiffness decreases noticeably, and the maximum tensile strength is significantly reduced compared with the non-aged samples, confirming the plasticization effect of the matrix and the weak fiber–matrix interface.

The statistical analysis of the sample sets for the plate with [0°/90°] fiber orientation, presented in Table 2, Table 3 and Table 4, was carried out using the same formulas in all cases.

The arithmetic mean of these four data points is the average ultimate tensile stress:

(8) $${\bar{\sigma }}_{UTS}=\frac{1023.16}{3}=341.05\;MPa$$

The sample standard deviation is

(9) $$S=\sqrt{\frac{\sum {\left(\sigma_{UTS}-{\bar{\sigma }}_{UTS}\right)}^{2}}{n-1}}$$

$$S=\sqrt{\frac{167.23}{2}}=9.14\;MPa$$

The coefficient of variation CV is defined as the ratio of the standard deviation S to the mean:

(10) $$CV=\frac{S}{{\bar{\sigma }}_{r}}$$

$$CV=\frac{9.14}{341.05}100=2.67\%$$

The mechanical properties of the immersed samples are summarized in Table 5.

**Figure 8:**
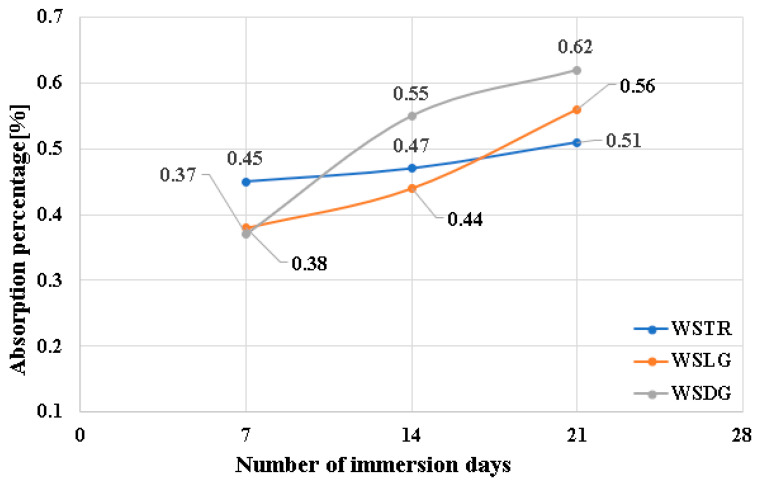
Variation in absorbed moisture depending on the number of days.

Figure 8 highlights the variation in water absorption percentage depending on immersion time for the three flow directions. In all three directions, a slow increase in the absorption percentage is observed from 7 to 21 days, indicating a process of water diffusion in the polymer matrix, characteristic of polymer matrix composite materials.

### 3.2. Three-Directional Impact Measurements on a GFRP Specimen Using a Drop Weight Machine

**Figure 9:**
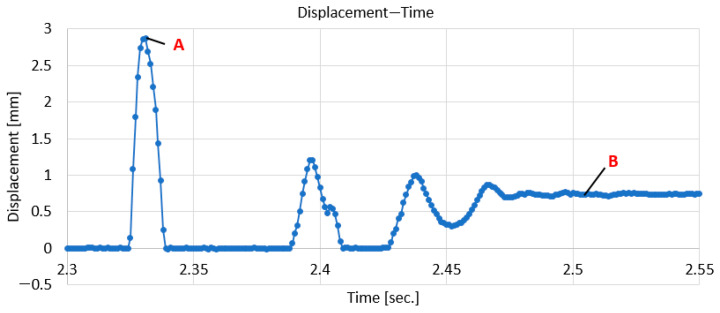
The variation in displacement in time, taken in the vicinity of the moment of impact.

Since the signals acquired from each sensor are similar for each of the 18 indentations, the signal areas with the variation in time in the vicinity of the moment of impact of the indenter on the composite sample are presented below. In Figure 9, the variation in the displacement at the moment of impact given by the displacement sensor located at the bottom of the plate, opposite the impact point, can be observed.

From Figure 9 it is observed that at the moment of impact, the point located on the surface opposite to the impacted ones (but in the same direction) moved by approximately 1.92 mm. Obviously, immediately after impact, there are still some oscillations of the contact point of the displacement sensor with the surface opposite to the impacted one. After the displacement is stabilized by the permanent deformation of the impacted area, it is found that the displacement stabilizes at approximately 0.7 mm. Under these conditions, an elastic recovery of approximately 1.22 mm is observed, but so is a residual deformation of 0.7 mm. These values differed from one impact to another, with those presented here being taken as an example only.

**Figure 10:**
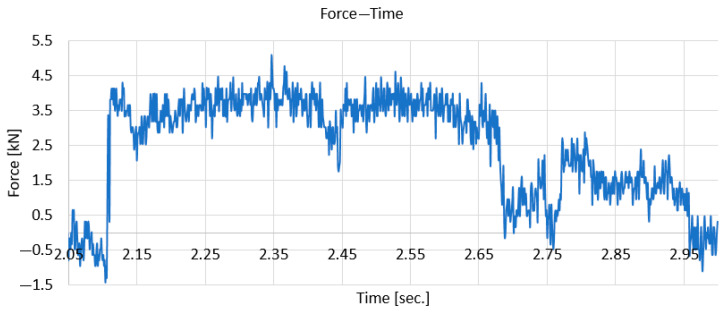
The variation in force over time, taken at the moment of impact.

Figure 10 shows the variation in the force in the period of time before and after the moment of impact.

It is found that the force increases suddenly from 0 to approximately 4 kN and is maintained for a short period of time (approx. 0.5 s) at this value, after which it decreases again to zero. The fluctuations visible in the figure are due to the high sampling rate (1000 Hz) and the fast response of the displacement sensor.

**Figure 11:**
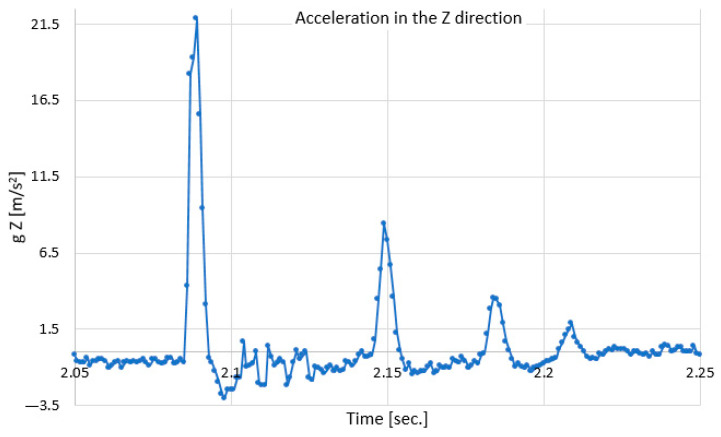
Variation in acceleration versus time in the Z (vertical) direction.

In Figure 11 we can see the variation in the acceleration in the vertical direction (Z) of the 8.2 kg mass falling from a height of 150 mm.

It can be seen that the maximum acceleration at the moment of impact is approx. 21.85 m/s^2^. Due to the subsequent displacements after the impact, the acceleration in the vertical direction shows successive increases and decreases until it returns to zero. Next, a series of graphs corresponding to the values acquired from the sensors placed on the test stand, in relation to the impact areas, are presented.

**Figure 12:**
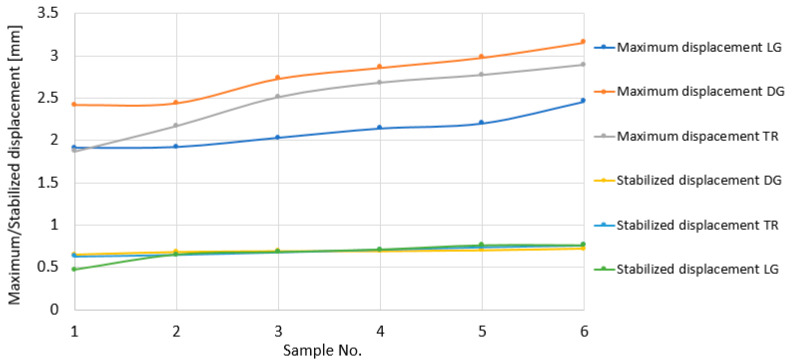
Total (maximum) and stabilized displacement graphs for LG, TR, and DG directions.

Figure 12 shows the variations in the maximum total displacements (point A in Figure 9) and those established after the impact (point B in Figure 9), obtained in the three directions. It is found that the highest values are obtained in the diagonal direction and the lowest values in the longitudinal direction. In the transverse direction, the maximum displacement values are between those in the diagonal and longitudinal directions. Closer values are obtained for the displacements of points 1(TR) and 1(LG) and points 6(TR) and 6(DG). On the other hand, it is found that the stabilized displacements, obtained after the impact had ceased, remain at approximately the same values.

**Figure 13:**
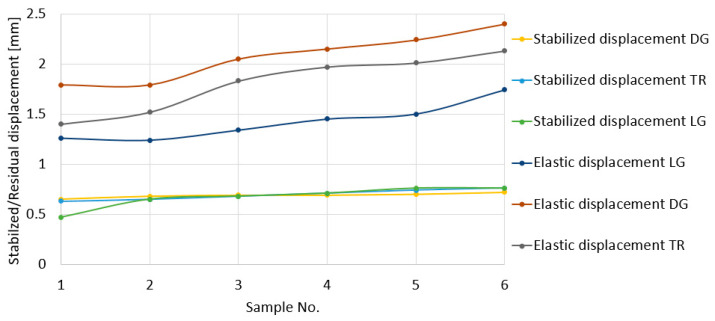
Elastic and stabilized displacement graphs for LG, TR, and DG directions.

Figure 13 shows the variation in the elastic displacement values in the three directions in which the impact was made. These were calculated as the difference between the maximum and stabilized displacements. Given that the stabilized displacement values remain approximately constant, it is found that the variation in the elastic displacements is proportional to that of the maximum displacements. As a result, here too, it is found that the highest values are obtained for the diagonal direction.

**Figure 14:**
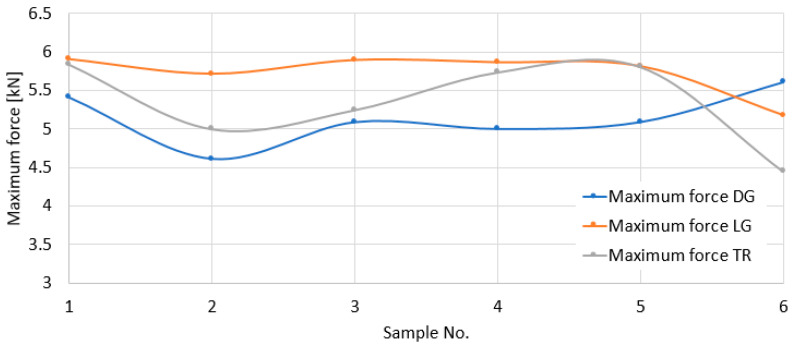
Graphs of maximum forces at impact for the LG, TR, and DG directions.

Figure 14 presents the graphs regarding the variation in the maximum forces obtained at the moment of impact, for the three impact directions.

It is found that there are no significant differences between the values of the maximum forces introduced as a result of the impact. However, the highest forces seem to be introduced with the impact in the longitudinal direction and the lowest ones with those in the diagonal direction. We recall that the highest displacements were recorded in the diagonal direction and the lowest ones in the longitudinal direction. This force–displacement comparison indicates that the higher displacements were not due to the higher forces (these being lower) but to the constitution of the material in relation to the impact area and the direction of the impact area.

**Figure 15:**
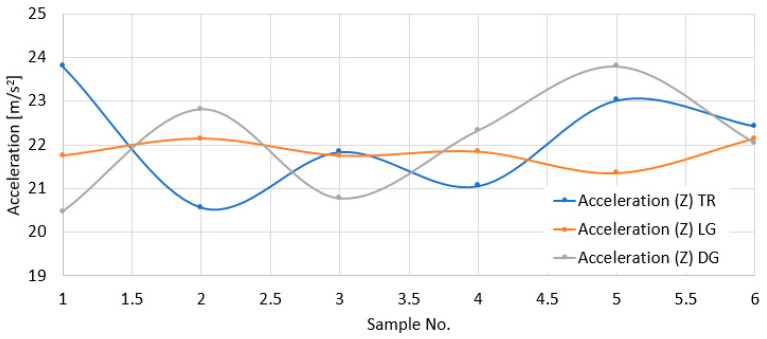
Maximum accelerations in the Z—vertical—direction for the LG, TR, and DG directions.

Figure 15 presents the acceleration values obtained at each impact point. The graphs were made for the three directions considered.

It is mentioned that the distances from the center of the indentations to the edge of the sample were the same for all the tests. The fact that the residual displacements obtained for the DG direction are the largest is explained in the following: The longitudinal and transverse reinforcement in the DG area was interrupted by the diagonal cut. Under these conditions, the structural possibilities of the material to resist the impact bending stress were lower than in the other two directions, where one of the reinforcements was cut at the edge but the other was intact.

### 3.3. DMA Measurements

**Figure 16:**
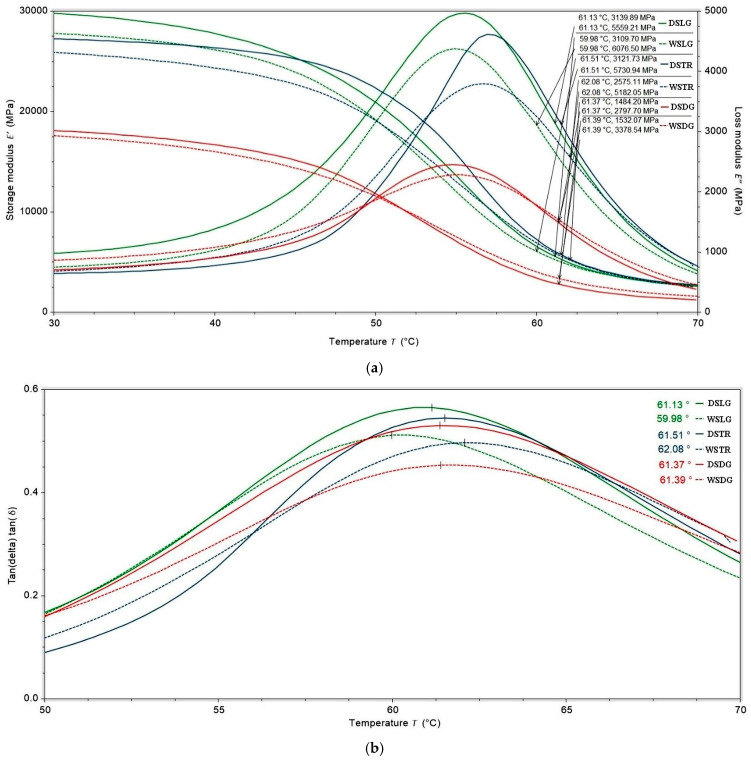
DMA test of GFRP sample: (**a**) storage modulus and loss modulus measured values for each Tg.t; (**b**) tan (δ) as a function of temperature with display of Tg values (Tg.t).

The viscoelastic temperature-dependent behavior of the epoxy–glass fiber composites was investigated by dynamic mechanical analysis measuring the storage modulus E′ and the loss modulus E″. The storage modulus is directly related to Young’s modulus and is considered to be a material’s capacity to store elastic energy, and the loss modulus quantifies the energy dissipated as heat, whereas the ratio of the latter to the former is regarded as the loss factor (tan δ). These temperature-dependent characteristics play a vital role in providing information on interfacial bonding between the reinforced fiber and the polymer matrix of a composite material. The influence of both water-immersion aging (samples were immersed in water for 7 days) and fiber orientation on the epoxy–glass fiber samples’ mechanical behavior was clearly observed, showing significant variations in terms of storage modulus, damping factor (tan δ), and glass transition temperature (Tg), as shown in Figure 16. The glass transition temperature Tg for each tested configuration was obtained by applying two common methods. The first method defines the temperature at the intersection point of the two tangent lines of the storage modulus curve as the Tg, which indicates the onset of degradation of the storage modulus and is denoted by Tg.0 [39]. Alternatively, the temperature at the maximum loss factor is often defined as the Tg, and it is denoted by Tg.t [40,41]. The corresponding values of both Tg.0 and Tg.t are given in Table 6. In this study, we employed the second method to obtain Tg values Tg.t for further analysis.

A significant influence of the fiber orientation on the epoxy–glass fiber samples’ stiffness was observed, while the effect on Tg was limited. This is consistent with the fact that fiber orientation does not directly affect the glass transition temperature of a composite, which is an intrinsic property of the polymer matrix itself. However, fiber orientation significantly impacts the material’s stiffness, strength, and thermal expansion. Moreover, the presence of voids and defects within the polymer structure can cause a weakening of the material, a reduction in interfacial contact leading to lower storage modulus, and a shift in the loss modulus and tan (δ) peaks to a lower temperature, as the presence of voids increases damping. The variation in storage modulus with fiber angle is shown in Figure 16a. Overall, the storage modulus values decreased with the increase in temperature, which is due to a reduction in the stiffness of the epoxy system analyzed. As shown in Figure 16a, at room temperature, the material in a glassy state, strongly packed, has low mobility and experiences strong intermolecular forces contributing to high modulus values; however, as the temperature increases, the molecular segments gain enough thermal energy to move, causing the material to transition from a brittle solid to a softer, rubbery state. Storage modulus values measured at room temperature for longitudinal (0°) and transversal (90°) samples are comparable and tend to reach nearly the same values when the temperature is increased to up to 70 °C. The reason is related to the bi-directional architecture of plain weave-woven glass made by interlacing two sets of yarns (warp and weft) at a 90-degree angle, where each weft yarn passes over one and under the next warp yarn. Although theoretically, in a plain weave, mechanical resistance is similar in the 0° and 90° directions, experimentally, there can still be a small degree of anisotropy. Likewise, as expected for the 45° fiber-reinforced epoxy composites, the storage modulus was significantly lower (45% reduction) compared with other samples because the load was applied at an angle with respect to the warp and weft yarns, causing the fibers to slide past each other and engage in shear, leading to lower strength and higher ductility. Nevertheless, overall, a significant decrease in the value of storage modulus, occurring at around 50° C for all curves, was observed, as shown in Figure 16a. This relates to the end of the glassy region for highly cross-linked thermoset polymers.

When the temperature was increased from 50 to around 65–70 °C, both 0° and 90° samples exhibited similar behavior in terms of stiffness, with comparable values of the storage modulus, while the 45°-aligned fiber orientation glass–epoxy composite retained the same decreasing trend, indicating a softer material configuration. The reason for this trend is that the stiffness of the specimen along the longitudinal direction relies on the alignment of fibers and decreases as the offset of the fiber axis increases, resulting in a reduction in bending resistance of the composite.

Furthermore, the influence of water-immersion aging on the glass transition temperature (Tg) was observed primarily in the 0° longitudinal and 90° transverse glass–epoxy composite samples, while it was much less pronounced in the 45° samples. The loss modulus variation in the glass–epoxy composite samples cut at different angles, 0°, 90°, and 45°, as a function of temperature is shown in Figure 16a. It is seen from the plots that the value of the loss factor increases with the rise in temperature, reaching a maximum value in the transition region and beginning to decrease in the rubbery area. The chain segments contained in the resin are in the frozen state below glass transition temperature Tg, causing low tan δ values in all laminates, whereas in the transition region, resin molecules achieve higher mobility, resulting in high tan δ values (Figure 16b). As observed in Figure 16, a declining trend in Tg is reported for the water-immersion-aged glass–epoxy matrix, regardless of orientation. A high magnitude of the peak in tan δ is observed for longitudinal (0°) and transversal (90°) samples in comparison with 45° samples for both aged and unaged samples. The shift in the peaks correlates with the reduction in Tg.

A significant decrease in the storage modulus (E′) in the glassy region indicates that load transfer from the matrix to the fibers is compromised, suggesting interfacial degradation in fiber-reinforced composites [42,43]. Concurrently, an increase in and broadening of the tan delta peak reflects higher internal friction and energy dissipation, which occurs when fibers slip or interact frictionally with a weakened matrix interface during oscillatory loading [44,45]. Together, these dynamic mechanical analysis (DMA) indicators provide reliable evidence of interface quality and viscoelastic behavior in polymer composites. These effects are most clearly observed in transverse specimens along the fiber direction.

The cross-link density of both water-immersion-aged and unaged epoxy–glass fiber-reinforced composite samples cut from the laminate in three different directions 0°, 90°, and 45° was determined using Equation (11) as follows and reported in Table 6

(11) $$\rho =\frac{G}{3RT^{\prime }}\;$$

where *ρ* is the cross-link density in mol/cm^3^, G refers to the storage modulus in MPa in the rubbery plateau region, R is the universal gas constant (8.3145 J/K mol), and T is the temperature in the rubbery plateau region in Kelvin at Tg + 50.

Table 6 reports the measured Tg (obtained via two methods), and the loss and storage modulus values, as well as the cross-link density calculated for both water-immersion-aged and unaged epoxy–glass fiber-reinforced composite samples cut from the laminate following three different directions, 0°, 90°, and 45°.

Water-immersion aging showed a general trend of reducing cross-link density and increasing molecular mobility, as shown in Table 6, regardless of fiber orientation. This decrease in molecular mobility can be attributed to a decrease in Tg. Higher values of cross-link density correspond to a higher storage modulus, as observed for longitudinal (0°) and transversal (90°) samples in comparison with 45° samples. Thus, the highly cross-linked polymer had a much higher storage modulus, demonstrating high stiffness and a close-fitting network structure, whereas the polymer with lower cross-link density demonstrated a lower storage modulus (45° samples).

The observed increase in effective cross-link density and storage modulus E′ in the water-immersion-aged samples, compared with the unaged specimens, can be explained by anti-plasticization effects, which mainly occur in the polymer matrix. During 7 days of exposure at room temperature, the polymer matrix absorbed a small amount of water, which occupied free volume and formed secondary hydrogen bonds with polar groups, thereby restricting segmental mobility. This restriction led to a temporary stiffening of the network, resulting in higher E′, without the formation of new covalent cross-links [46]. This mechanism provides a plausible explanation for the modest increases in mechanical properties observed in the aged composite samples.

### 3.4. TMA Measurements

**Figure 17:**
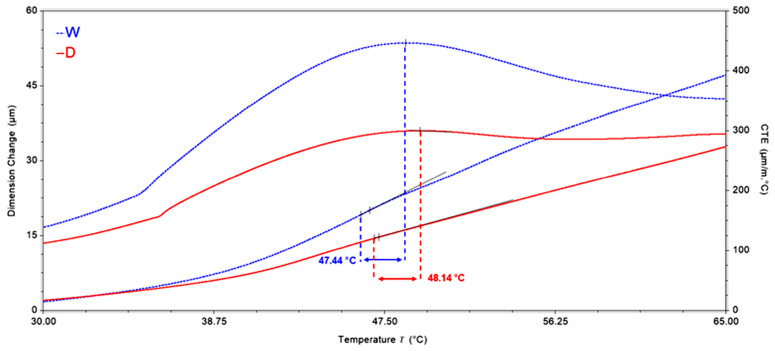
Dimension change as a function of temperature for aged (W) and unaged (D) glass–epoxy composites.

TMA analysis were used to determine the coefficient of thermal expansion (CTE) and glass transition temperature (Tg). The change in slope shown in Figure 17, displaying the dimension variation as a function of temperature for aged and unaged glass–epoxy composites, indicates a Tg very similar to the Tg.0 obtained using DMA. Figure 17 shows an example where the end of the major dimension change is the peak of the derivative (49.32 °C for unaged and 48.59 °C for aged glass–epoxy composites) and the onset is the change in the slope of the derivative (46.97 °C for unaged and 46.26 °C for aged glass–epoxy composites). The Tg was then taken to be the midpoint of these two measurements (in this case, 48.14 °C for unaged and 47.44 °C for aged glass–epoxy composites). This technique enables to obtain a consistent measurement of Tg, similar to the value of Tg.0, determined at the intersection point of the two tangent lines of the storage modulus curve.

A regression model was used for the calculation of the coefficient of linear thermal expansion (Equation (7)), and the results obtained are compared in Table 7.

## 4. Conclusions

This paper presents the experimental results of a research study on a bi-directional fabric-reinforced composite. The mechanical behavior and properties of GFRP which was subjected to tension, impact, DMA, and TMA tests are presented.

Within the tensile loading regime, tests were carried out along three directions (TR, LG, and DG), highlighting the variation in specific mechanical characteristics. The comparison between dry and immersed specimens represents a relevant criterion for assessing the degradation of GFRP materials under different environmental conditions. In the case of impact loading, the novelty of the study lies in evaluating damage as a function of the reinforcement directions. The proximity to the cut edge—where either the weft, the warp, or both were interrupted—significantly influenced the impact response. The influence of specimen edges was investigated in the impact tests, thus simulating the types of impacts that wind turbine blades may experience from hailstones or wind-borne particles in the leading-edge or trailing-edge regions. DMA and TMA tests revealed clear differences in terms of elasticity modulus and thermal expansion coefficient between dry samples and those conditioned under humidity conditions. Similar to the static tensile loading case, these analyses highlight the degradation of material properties induced by environmental exposure.

Based on the results obtained, the following can be concluded:

1. The experimental results confirm the pronounced orthotropic behavior of the GFRP composite, with mechanical properties being strongly dependent on fiber orientation and matrix contribution.
2. Longitudinally cut test specimens exhibited the highest tensile strength values, with predominantly elastic behavior.
3. Water-immersion aging led to progressive deterioration of mechanical properties for all cutting directions, with the most pronounced reduction in tensile strength being observed after 21 days of immersion, due to degradation of the fiber–matrix interface.
4. Impact testing revealed higher maximum and elastic displacements in the diagonal direction, while the highest impact forces were recorded in the longitudinal direction, indicating increased stiffness along the fiber axis; acceleration values showed greater dispersion in the diagonal and transverse directions.
5. The effect of both water-immersion aging and fiber orientation on the macroscopic mechanical behavior of epoxy–glass fiber samples was clearly observed, showing significant dissimilarities in terms of storage modulus damping factor (tan δ) and glass transition temperature (Tg).
6. DMA analysis showed a shift in Tg when changing the fiber direction from longitudinal (0°) and transversal (90°) to 45°, with a visible decrease in aged samples. A similar decreasing trend was observed in storage modulus and cross-link density. However, the 45° orientation exhibited a particular behavior potentially due to the presence of voids in the polymer structure, which decrease interfacial contact, leading to lower storage modulus and a shift in the loss modulus and tan (δ) peaks to a lower temperature, as the presence of voids increases damping.
7. TMA analysis demonstrated a significant increase in the coefficient of thermal expansion after water-immersion aging, attributed to matrix plasticization, with implications for numerical modeling and durability assessment of GFRP structures such as wind turbine blades and drones.

## Figures and Tables

**Table 1:** Characteristics of epoxy resin of type IN2.

Property	Unit	Value
Density	[g/cm^3^]	1.08–1.12
Hardness	Shore D	84–88
Maximum Tg	°C	92–98
Flexural strength	[MPa]	110–120
Flexural modulus	[MPa]	3100–3500
Tensile strength	[MPa]	68.5–76
Compressive strength	[MPa]	88–100
Elongation of break	[%]	6–8
Maximum strain	[%]	5–7
Water absorption at 23 °C in 24 h	[%]	0.10–0.20

**Table 2:** Standard deviation for transversely cut specimens.

Sample No.	σ_UTS_ [MPa]	${\bar{\sigma }}_{UTS}$ [MPa]	Deviations from the Mean $\sigma_{r}-{\bar{\sigma }}_{r}$ [MPa]	${\left(\sigma_{r}-{\bar{\sigma }}_{r}\right)}^{2}$ [MPa]	Standard Deviation S [MPa]	Coefficient of Variation CV [%]
1	334.5	341.05	−6.55	42.90	9.14	2.67
2	351.5		10.45	109.20		
3	337.16		−3.89	15.13		
Σ=	1023.16			167.23		

**Table 3:** Standard deviation for longitudinally cut specimens.

Sample No.	σ_UTS_ [MPa]	${\bar{\sigma }}_{UTS}$ [MPa]	Deviations from the Mean $\sigma_{r}-{\bar{\sigma }}_{r}$ [MPa]	${\left(\sigma_{r}-{\bar{\sigma }}_{r}\right)}^{2}$ [MPa]	Standard Deviation S [MPa]	Coefficient of Variation CV [%]
1	369.16	340.60	28.56	815.67	35.64	10.46
2	352		11.4	129.96		
3	300.66		−39.94	1595.20		
Σ=	1021.82			2540.83		

**Table 4:** Standard deviation for diagonally cut specimens.

Sample No.	σ_UTS_ [MPa]	${\bar{\sigma }}_{UTS}$ [MPa]	Deviations from the Mean $\sigma_{r}-{\bar{\sigma }}_{r}$ [MPa]	${\left(\sigma_{r}-{\bar{\sigma }}_{r}\right)}^{2}$ [MPa]	Standard Deviation S [MPa]	Coefficient of Variation CV [%]
1	90.83	83.38	7.45	55.50	3.99	4.78
2	82.16		−1.22	1.48		
3	77.16		−6.22	38.68		
Σ=	250.15			31.88		

**Table 5:** Mechanical properties of aged samples.

Sample WS	σ_UTS_-TR [MPa]	σ_UTS_-LG [MPa]	σ_UTS_-DG [MPa]
WS7	249.83	243.16	72.66
WS14	231.5	237.16	64.5
WS21	207.5	229.16	63.83

**Table 6:** DMA parameters and cross-link densities of aged and unaged glass–epoxy composites.

Sample	Tg.0 [°C]	Tg.t [°C]	Storage Modulus E′ [MPa]	Loss Modulus E″ [MPa]	Cross-Link Density [×10^−2^ mol/cm^3^]
DSLG	46.48	61.13	3139.89	5559.21	0.3275
WSLG	45.76	59.98	3109.70	6076.50	0.3253
DSTR	49.72	61.51	3121.73	5730.94	0.3253
WSTR	46.77	62.08	2575.11	5182.05	0.2679
DSDG	45.04	61.37	1484.20	2797.70	0.1547
WSDG	45.22	61.39	1532.07	3378.54	0.1597

**Table 7:** CTE of water-immersion-aged (W) and unaged (D) glass–epoxy composites.

Sample	Coefficient of Thermal Expansion	
	Below Tg [30–Tg °C]	Above Tg [Tg–65 °C]
W	291.65 µm/(m·°C)	398.7 µm/(m·°C)
D	205.6 µm/(m·°C)	296.75 µm/(m·°C)

## Data Availability

The data presented in this study are available upon request from the corresponding author.

## Abbreviations

The following abbreviations are used in this manuscript:

GFRP	Glass fiber-reinforced polymer
DMA	Dynamical mechanical analysis
TMA	Thermomechanical analysis
LG	Longitudinal
TR	Transversal
DG	Diagonal
WS	Water specimen
DMAD	Dynamical mechanical analysis—dry
DMAW	Dynamical mechanical analysis—water
TMAD	Thermomechanical analysis—dry
TMAW	Thermomechanical analysis—water
IS	Impact specimen
DS	Dry specimen

## References

[1] Rajak D.K., Pagar D.D., Menezes P.L., Linul E. (2019). Fiber-Reinforced Polymer Composites: Manufacturing, Properties, and Applications. Polymers.

[2] Essam S., Ahmad W. Manufacturing Cost Modelling for Aerospace Composite Applications. Proceedings of the 19th ISPE International Conference on Concurrent Engineering—CE.

[3] Petersson H., Motte D., Bjärnemo R. Carbon Fiber Composite Materials in Modern Day Automotive Production Lines: A Case Study. Proceedings of the ASME International Mechanical Engineering Congress and Exposition.

[4] Rubino F., Nisticò A., Tucci F., Carlone P. (2020). Marine Application of Fiber Reinforced Composites: A Review. J. Mar. Sci. Eng..

[5] Karbhari V.M., Zhao L. (2000). Use of composites for 21st century civil infrastructure. Comput. Methods Appl. Mech. Eng..

[6] Osswald T.A., David J., Thompson M.S. (2022). Polymer composites: Additive manufacturing of composites. Polym. Compos..

[7] Huang X., Su S., Xu Z., Miao Q., Li W., Wang L. (2023). Advanced Composite Materials for Structure Strengthening and Resilience Improvement. Buildings.

[8] Stanciu M.D., Drăghicescu H.T., Rosca I.C. (2021). Mechanical Properties of GFRPs Exposed to Tensile, Compression and Tensile–Tensile Cyclic Tests. Polymers.

[9] Stanciu M.D., Savin A., Drăghicescu H.T. (2017). The Assessing of the Failure Behavior of Glass/Polyester Composites Subject to Quasi Static Stresses. Mater. Sci. Eng..

[10] Drăghicescu H.T., Vlase S., Stanciu M.D., Curtu I., Mihalcica M. (2015). Advanced Pultruded Glass Fibers-Reinforced Isophtalic Polyester Resin. Mater. Plast.

[11] Erden S., Sever K., Seki Y., Sarikanat M. (2010). Enhancement of the mechanical properties of glass/polyester composites via matrix modification glass/polyester composite siloxane matrix modification. Fibers Polym..

[12] Lemaitre J., Chaboche L. (1994). Mechanics of Solid Materials.

[13] Slamani M., Châtelain J.F. (2023). A review on the machining of polymer composites reinforced with carbon (CFRP) glass (GFRP), and natural fibers (NFRP). Discov. Mech. Eng..

[14] He H., Gao F., Li L. (2013). Effect of epoxy resin properties on the mechanical properties of carbon fiber/epoxy resin composites. Int. J. Mater. Res..

[15] Jeong J., Lee Y.H., Park K.T., Hwang Y.K. (2007). Field and laboratory performance of a rectangular shaped glass fiber reinforced polymer deck. Compos. Struct..

[16] Adekomaya O., Adama K. (2017). Glass-fibre reinforced composites: The effect of fibre loading and orientation on tensile and impact strength. Niger. J. Technol..

[17] Cerbu C., Cosereanu C. (2016). Moisture Effects on the Mechanical Behavior of FirWood Flour/Glass Reinforced Epoxy Composite. BioResources.

[18] Eftekhari M., Fatemi A. (2016). On the strengthening effect of increasing cycling frequency on fatigue behavior of some polymers and their composites: Experiments and modeling. Int. J. Fatigue.

[19] Patel V.K., Rawat N. (2017). Physico-mechanical properties of sustainable Sagwan-Teak Wood Flour/Polyester Composites with/without gum rosin. Sustain. Mater. Technol..

[20] Torabizadeh M.A. (2013). Tensile, compressive and shear properties of unidirectional glass/epoxy composite subjected to mechanical loading and low temperature services. Indian J. Eng. Mater. Sci..

[21] Tang H., Nguyen T., Chuang T., Chin J., Lesko J., Wu F. (2000). Fatigue Model for fiber-reinforced polymeric composite. J. Mater. Civ. Eng..

[22] Steigmann R., Savin A., Goanta V., Barsanescu P.D., Leitoiu B., Iftimie N., Stanciu M., Curtu I. (2016). Determination of mechanical properties of some glass fiber reinforced plastics suitable to Wind Turbine Blade construction. IOP Conf. Ser. Mater. Sci. Eng..

[23] Selzer R., Friedrich K. (1997). Mechanical properties and failure behavior of carbon fibre-reinforced polymer composites under the influence of moisture. Compos. Part A.

[24] Huang G., Sun H. (2007). Effect of water absorption on the mechanical properties of glass/polyester composites. Mater. Des..

[25] Hussnain S.M., Shah S.Z.H., Megat-Yusoff P.S.M., Hussain M.Z. (2023). Degradation and mechanical performance of fibre-rei forced polymer composites under marine environments: A review of recent advancements. Polym. Degrad. Stab..

[26] Wang P., Ke L., Wu H., Leung C.K.Y., Li L. (2023). Hygrothermal aging effects on the diffusion-degradation process of GFRP composite: Experimental study and numerical simulation. Constr. Build. Mater..

[27] Rosa I.C., Firmo J.P., Correia J.R., Mazzuca P. Influence of elevated temperatures on the bond behaviour of GFRP bars to concrete—Pull-out tests. Proceedings of the IABSE Symposium, Guimaraes 2019: Towards a Resilient Built Environment Risk and Asset Management—Report.

[28] Safri S.N.A., Sultan M.T.H., Aminanda Y. (2014). Impact Characterisation of Glass Fibre Reinforced Polymer (GFRP) Type C-600 and E-800 Using a Drop Weight Machine. Appl. Mech. Mater..

[29] Safri S.N.A., Sultan M.T.H., Cardona F. (2015). Impact damage evaluation of glass-fiber reinforced polymer (GFRP) using the drop test rig—An experimental based approach. ARPN J. Eng. Appl. Sci..

[30] IN2 Epoxy Resin. https://media.easycomposites.eu/datasheets/EC-TDS-IN2-Infusion-Resin.pdf.

[31] (2018). Standard Test Method for Tensile Properties of Polymer Matrix Composite Materials.

[32] (2020). Standard Test Method for Moisture Absorption Properties and Equilibrium Conditioning of Polymer Matrix Composite Materials.

[33] (2020). Standard Test Method for Measuring the Damage Resistance of a Fiber-Reinforced Polymer Matrix Composite to a Drop-Weight Impact Event.

[34] (2024). Standard Test Method for Glass Transition Temperature (DMA Tg) of Polymer Matrix Composites by Dynamic Mechanical Analysis (DMA).

[35] (2025). Standard Test Method for Linear Thermal Expansion of Solid Materials by Thermomechanical Analysis.

[36] Lakshmanan M., Jayanarayanan K., Joesph J. An Experimental Investigation of Fracture Toughness and Volume Resistivity of Symmetric Laminated Epoxy/Glass Fiber/CNT multiscale composites. Proceedings of the International Conference on Advances in Materials and Manufacturing Applications (IConAMMA-2018).

[37] Wu L., Adam L., Doghri I., Noels L. (2017). An incremental-secant mean-field homogenization method with second statistical moments for elasto-visco-plastic composite materials. Mech. Mater..

[38] Czarnota C., Kowalczyk-Gajewska K., Salahouelhadj A., Martiny M., Mercier S. (2015). Modeling of the cyclic behavior of elastic– viscoplastic composites by the additive tangent Mori–Tanaka approach and validation by finite element calculations. Int. J. Solids Struct..

[39] Michels J., Widmann R., Czaderski C., Allahvirdizadeh R., Motavalli M. (2015). Glass transition evaluation of commercially available epoxy resins used for civil engineering applications. Compos. Part B.

[40] Korayem H.A., Chen S.J., Zhang Q.H., Li Y.C., Zhao L.X., Duan H.D. (2016). Failure of CFRP-to-steel double strap joint bonded using carbon nanotubes modified epoxy adhesive at moderately elevated temperatures. Compos. Part B.

[41] Korayem H.A., Barati R.M., Simon G.P., Zhao L.X., Duan H.W. (2014). Reinforcing brittle and ductile epoxy matrices using carbon nanotubes masterbatch. Compos. Part A.

[42] Afaghi-Khatibi A., Mai Y.-W. (2002). Characterisation of fibre/matrix interfacial degradation under cyclic fatigue loading using dynamic mechanical analysis. Compos. Part A Appl. Sci. Manuf..

[43] Bashir M.A. (2021). Use of Dynamic Mechanical Analysis (DMA) for Characterizing Interfacial Interactions in Filled Polymers. Solids.

[44] Shen Y., Tan J., Fernandes L., Qu Z., Li Y. (2019). Dynamic Mechanical Analysis on Delaminated Flax Fiber Reinforced Composites. Materials.

[45] Venkategowda T., Manjunatha L.H., Anilkumar P.R. (2022). Dynamic mechanical behavior of natural fibers reinforced polymer matrix composites—A review. Mater. Today Proc..

[46] Mascia L., Kouparitsas Y., Nocita D., Bao X. (2020). Antiplasticization of Polymer Materials: Structural Aspects and Effects on Mechanical and Diffusion-Controlled Properties. Polymers.

